# Identifying Mechanisms of Normal Cognitive Aging Using a Novel Mouse Genetic Reference Panel

**DOI:** 10.3389/fcell.2020.562662

**Published:** 2020-09-11

**Authors:** Amy R. Dunn, Niran Hadad, Sarah M. Neuner, Ji-Gang Zhang, Vivek M. Philip, Logan Dumitrescu, Timothy J. Hohman, Jeremy H. Herskowitz, Kristen M. S. O’Connell, Catherine C. Kaczorowski

**Affiliations:** ^1^The Jackson Laboratory, Bar Harbor, ME, United States; ^2^Department of Anatomy and Neurobiology, The University of Tennessee Health Science Center, Memphis, TN, United States; ^3^Vanderbilt Memory and Alzheimer’s Center and Vanderbilt Genetics Institute, Vanderbilt University Medical Center, Nashville, TN, United States; ^4^Center for Neurodegeneration and Experimental Therapeutics and Department of Neurology, The University of Alabama at Birmingham, Birmingham, AL, United States

**Keywords:** cognitive aging, cognitive reserve, cognitive resilience, Weighted Gene Co-expression Network Analysis, quantitative trait locus mapping, Y-maze, contextual fear conditioning

## Abstract

Developing strategies to maintain cognitive health is critical to quality of life during aging. The basis of healthy cognitive aging is poorly understood; thus, it is difficult to predict who will have normal cognition later in life. Individuals may have higher baseline functioning (cognitive reserve) and others may maintain or even improve with age (cognitive resilience). Understanding the mechanisms underlying cognitive reserve and resilience may hold the key to new therapeutic strategies for maintaining cognitive health. However, reserve and resilience have been inconsistently defined in human studies. Additionally, our understanding of the molecular and cellular bases of these phenomena is poor, compounded by a lack of longitudinal molecular and cognitive data that fully capture the dynamic trajectories of cognitive aging. Here, we used a genetically diverse mouse population (B6-BXDs) to characterize individual differences in cognitive abilities in adulthood and investigate evidence of cognitive reserve and/or resilience in middle-aged mice. We tested cognitive function at two ages (6 months and 14 months) using y-maze and contextual fear conditioning. We observed heritable variation in performance on these traits (*h*^2^_*RIx̄*_ = 0.51–0.74), suggesting moderate to strong genetic control depending on the cognitive domain. Due to the polygenetic nature of cognitive function, we did not find QTLs significantly associated with y-maze, contextual fear acquisition (CFA) or memory, or decline in cognitive function at the genome-wide level. To more precisely interrogate the molecular regulation of variation in these traits, we employed RNA-seq and identified gene networks related to transcription/translation, cellular metabolism, and neuronal function that were associated with working memory, contextual fear memory, and cognitive decline. Using this method, we nominate the *Trio* gene as a modulator of working memory ability. Finally, we propose a conceptual framework for identifying strains exhibiting cognitive reserve and/or resilience to assess whether these traits can be observed in middle-aged B6-BXDs. Though we found that earlier cognitive reserve evident early in life protects against cognitive impairment later in life, cognitive performance and age-related decline fell along a continuum, with no clear genotypes emerging as exemplars of exceptional reserve or resilience – leading to recommendations for future use of aging mouse populations to understand the nature of cognitive reserve and resilience.

## Introduction

Cognitive decline with age, even in the absence of overt dementia, is common and highly heritable (Dutta et al., 2014; Reynolds and Finkel, 2015). Cognitive function in old age is an important predictor of quality of life (Pan et al., 2011, 2015), and developing strategies to improve cognitive longevity (i.e., ability to maintain high level of cognitive function into old age) will be critical as life expectancy continues to increase through modern medicine. To understand cognitive stability in aging, it is important to first consider baseline cognitive function: namely, knowing individuals’ baseline function in early adulthood is necessary to fully capture cognitive aging trajectories, and understanding how baseline cognitive function is regulated may help to inform strategies to maintain those cognitive abilities in aging. Recent studies have identified over 300 loci associated with general cognitive function and related traits in adulthood (Davies et al., 2011, 2015, 2018; Hill et al., 2014; Hibar et al., 2015, 2017; Trampush et al., 2015; Clarke et al., 2016; Krapohl and Plomin, 2016; Okbay et al., 2016; Sniekers et al., 2017; Savage et al., 2018; Zabaneh et al., 2018). Given the lack of longitudinal molecular data, and to a lesser extent, longitudinal cognitive data from human populations, it remains unclear if the mechanisms underlying baseline cognitive function also mediate normal cognitive aging. These factors are highly complex and poorly understood, despite extensive study (Harris and Deary, 2011; Bis et al., 2012; De Jager et al., 2012; Davies et al., 2014; Mukherjee et al., 2014; Zhang and Pierce, 2014; Debette et al., 2015; Lu et al., 2017; Raj et al., 2017; Tasaki et al., 2018; Yen et al., 2018; Kamboh et al., 2019; Wingo et al., 2019). Discovering high-impact targets for bolstering baseline cognitive function and enhancing cognitive longevity will facilitate the development of pharmacotherapeutics to enhance cognitive health in middle-age and beyond.

Identifying molecular networks that promote the maintenance of cognitive function in aging requires understanding of cognitive reserve and resilience. Cognitive reserve is often defined as higher baseline function (Montine et al., 2019), whereas cognitive resilience is characterized by slower cognitive decline. Reserve and resilience have often been attributed to environmental factors; for example, socioeconomic status, education level, and physical activity are all associated with greater cognitive reserve and better cognitive status in late adulthood (Arenaza-Urquijo et al., 2015; Walhovd et al., 2019; Zahodne et al., 2019). However, given the heritability of cognitive decline [∼30–60% genetic control based on twin and community studies (Swan et al., 1990; McGue and Christensen, 2001; Harris and Deary, 2011)], there is also a significant genetic component. Perhaps unsurprisingly, molecular pathways that have been implicated in mediating human cognitive aging and reserve include synaptic function (Honer et al., 2012; Arenaza-Urquijo et al., 2015; Lesuis et al., 2018; Kamboh et al., 2019; Wingo et al., 2019), mitochondrial function (Wingo et al., 2019), and inflammation (Stacey et al., 2017).

In order to fully understand molecular contributors to cognitive aging, it will be necessary to study transcriptomic, proteomic and epigenetic changes across the lifespan and how they relate to and predict changes in cognitive function. Human studies of aging often recruit participants in middle age and necessarily collect brain tissue postmortem, at which point molecular signatures of those processes underlying the onset and progression of cognitive aging may have been ongoing for decades. Even studies that begin sampling cognitive function earlier in life are unable to capture molecular changes within the brain until after death, which precludes the possibility of understanding early molecular regulators of cognitive reserve and resilience.

Given the challenges in studying the genetics of cognitive decline in humans, animal models of aging provide a unique and critical opportunity to study molecular mechanisms of cognitive aging, as well as cognitive reserve and resilience, across the lifespan. In this study, we utilized a novel genetic reference population, an F1 population of C57BL/6J (B6) mice crossed with 27 strains of the BXD genetic reference panel of mice (B6-BXD), to interrogate molecular mediators of baseline cognitive function and age-related cognitive decline. The advantage of working with this population of mice is: (1) a well characterized, diverse and replicable genome, (2) the ability to sample a range of cognitive domains in both longitudinal and cross-sectional manners, (3) the availability of postmortem brain tissue at multiple ages for assessing gene and protein expression, and (4) the enhanced ability to identify genetic factors in the B6 genome that may confer protection against age-related decline. With this panel, we are able to take advantage of testing reproducible genotypes in controlled environments to study how age interacts with genetic background to influence cognitive decline. As in humans, we found that individual differences in cognitive function and changes across the lifespan are highly heritable and polygenetic in nature. To reveal the underlying molecular mechanisms, we performed RNA sequencing and identified gene co-expression networks involving intracellular, organelle and neuronal function whose expression profiles were strongly associated with cognitive function and cognitive aging. Finally, we evaluated operational definitions for cognitive reserve, resilience and reserve/resilience that we pre-registered to assess whether any B6-BXD strains exemplify cognitive reserve and resilience. From this work, we provide recommendations for incorporating genetically diverse, recombinant inbred mouse populations for aging studies and developing definitions of reserve and resilience for animal studies, as these are needed to advance our understanding of the mechanisms of reserve and resilience.

## Materials and Methods

### Animals

Animals were kept on a 12 h light/dark cycle and provided food and water *ad libitum*. Mice were group-housed (2–5 per cage). All routine procedures were approved by the Institutional Animal Care and Use Committee (IACUC) at The University of Tennessee Health Science Center, and in accordance with the standards of the Association for the Assessment and Accreditation of Laboratory Animal Care (AAALAC) and the National Institutes of Health Guide of the Care and Use of Laboratory Animals.

#### Generation of Ntg B6-BXD F1 Panel

Non-transgenic littermates of the AD-BXD panel were generated as described in Neuner et al. (2019). Briefly, hemizygous 5XFAD female mice on a congenic C57BL/6J background were crossed to male BXD mice (27 BXD strains). One-half of the resultant F1 offspring harbored the 5XFAD transgene to represent a familial Alzheimer’s disease population (AD-BXDs). The remaining half of the resultant F1 offspring did not inherit the 5XFAD transgene and were thus “normal aging” controls. Data collected from non-transgenic F1 population (B6-BXD) were comprehensively analyzed and interpreted here, though some behavioral and molecular data from these non-transgenic littermates was made available to the research community as controls for the AD-BXD panel first reported in Neuner et al., 2019. Because we had more thorough representation of female animals, we focused our behavioral and transcriptomic analyses on females only for this manuscript.

### Behavioral Analysis, Phenotype Derivation

**FIGURE 1:**
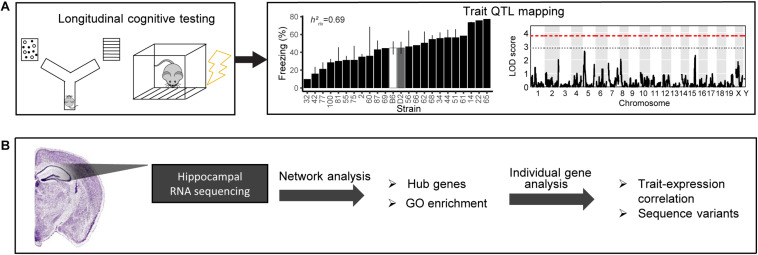
Schematic of our pipeline to identify mechanisms underlying cognitive function and aging. **(A)** We cognitively tested a large cohort of B6-BXD mice using y-maze (working memory) and contextual fear conditioning (short- and long-term memory). We performed quantitative trait locus (QTL) mapping on all traits. **(B)** We also performed bulk RNA sequencing of the hippocampus to identify molecular candidates mediating age-related cognitive decline. We analyzed transcriptomic data on a network level to identify gene networks underlying cognitive traits and age-related decline, as well as gene ontology (GO) terms summarizing the function of those networks. We also performed individual gene analysis, including identifying network hub genes and genes with high-impact sequence variants to prioritize genes within networks that underlying variation in cognitive decline.

Behavioral tasks (Figure 1A) were described in Neuner et al., 2019; a subset of these animals (i.e., all female non-transgenic animals) are described here. A total of 192 animals were included in the present analyses, and these animals numbers per strain, age, and assay may be found in Supplementary Table S1. Y-maze was conducted at both 6 months (*n* = 171) and 14 months (*n* = 100), with animals in the 14 months cohort having been previously tested at 6 months. The same cohort underwent contextual fear conditioning as a terminal assay at either 6 months (*n* = 83) or 14 months (*n* = 106). Brief descriptions of each phenotyping task are described below.

#### Y-Maze

To assess working memory function, mice were placed in a clear acrylic Y-maze for 8 min. External visual spatial cues were placed approximately one foot outside of the maze. Mouse movement was recorded with a video camera and spontaneous alternations were tracked by Any-Maze software. Spontaneous alternations were defined as successive entries into each arm before re-entering any arm. Chance performance was defined as less than 50% correct spontaneous alternations. Animal order was randomized, and experimenters were blind to mouse strain, age, and genotype.

#### Contextual Fear Conditioning

To assess contextual fear acquisition (CFA) and long-term contextual memory (CFM), animals underwent a standard contextual fear conditioning paradigm (Neuner et al., 2015). Training consisted of a 150 s baseline period followed by four footshocks (1 s, 0.9 mA) separated by 140 ± 5 s. A 40 s period following each shock was considered the postshock (PS) interval. Total freezing during the fourth PS interval (PS4) was defined as CFA. To measure CFM, animals were placed in the same chamber 24 h later for 10 min with no footshocks. Percent time spent freezing during training and testing was determined using FreezeFrame software.

#### Decline Scores

Age-related decline on memory function was determined by subtracting performance at 6 months from performance at 14 months to achieve a strain average “decline score” in each memory assay.

### Heritability Calculations

Heritability of behavioral phenotypes was calculated as a ratio of genetic variance to total variance (genetic + environmental variance), normalized to the number of biological replicates per strain (i.e., *h*^2^_*RIx̄*_; Figure 1A). Heritability scores can range from 0 to 1.0, with an *h*^2^_*RIx̄*_ = 1.0 indicating that 100% of the variance in that trait is controlled by genetics (Belknap, 1998).

### Trait and Module QTL Mapping

Genotypes for BXD strains were obtained from GeneNetwork.org. Quantitative trait locus (QTL) mapping was performed using the R package qtl2 (Broman et al., 2019) using the LOCO method for kinship correction (Figure 1A). Permutation tests (1,000) were used to determine statistical significance. Power calculations and percent variance explained were calculated using the R package qtlDesign and reported in Table 1.

**TABLE 1:** Heritability and QTL power calculation results for each cognitive trait.

Trait	*h*^2^_*RIx̄*_	Peak marker SNP	Power	Minimum percent variance explained by peak marker
Y-maze (6 months)	0.51	rs29776171	0.82	44.2%
Y-maze (14 months)	0.62	rs29525970	0.84	47.1%
CFA (6 months)	0.69	rs31878001	0.007	6.3%
CFA (14 months)	0.68	rs215717346	0.95	56.4%
CFM (6 months)	0.64	Affy_PC2_15	0.94	53.9%
CFM (14 months)	0.74	Affy_17539964	0.87	51.4%

### RNA Sequencing

RNA sequencing data from the present cohort has been previously reported in part in Neuner et al. (2019). The previous publication focused primarily on RNA expression in the 5XFAD-positive transgenic littermates (AD-BXDs) of the non-transgenic mice included herein, with expression data presented for certain genes of interest in transgenic mice in relation to non-transgenic mice. Here, we focus on the female non-transgenic B6-BXD mice only and the gene networks relevant to normal cognition and cognitive aging (Figure 1B). Sequencing methods were also described in Neuner et al. (2019). Briefly, hippocampi were snap-frozen at 6 m and 14 m (*n* = 39 for 6 months, *n* = 45 for 14 months) immediately following contextual fear conditioning, and RNA was isolated using a Qiacube and RNeasy Mini kit (Qiagen). Libraries were prepared using Truseq Stranded mRNA Sample Preparation Kit and sequenced by 75 bp paired-end sequencing on an Illumina HiSeq2500. We aligned reads from the non-transgenic female cohort to a diploid B6/D2 transcriptome using the EMASE pipeline (Raghupathy et al., 2018). Genes were filtered to require an average of at least 1 transcript per million in 50% of the samples and averaged across age and strain for downstream analyses. 15,327 genes survived this filter and were included in our final analyses.

#### Gene Co-expression Network Analysis

Co-expressed gene modules were generated from 6-months, 14 months, and population-wide female non-transgenic B6-BXD RNA-seq data by Weighted Gene Co-expression Network Analysis (WGCNA) (Langfelder and Horvath, 2008). A minimum module size of 30 was implemented, and the modules were assembled by block-wise network construction. In this study, the power β with scale-free *R*^2^ > 0.80 was adopted as a soft-thresholding index to construct a scale-free co-expression network. Module eigengene expression values from each module were used for downstream analyses.

#### Hub Gene Analysis

Module hub genes were defined as the gene with the highest connectivity in each module and identified using the function “*chooseTopHubInEachModule*” in the WGCNA R package.

#### Trait-Expression Correlations (Figure 1B)

To identify modules that were significantly associated with each given trait, we calculated the Pearson’s correlation coefficient of the module eigengene with each cognitive trait. Co-expression modules were identified as showing significant associations with a trait with an FDR < 0.05.

#### Co-expression Module Characterization (Figure 1B)

To characterize genes within our WGCNA modules, we performed functional enrichment analysis using the R package anRichment. Results were filtered to include only “Biological process” and “Molecular function” GO terms. FDR < 0.05 was used as the threshold to identify GO terms/pathways significantly enriched within each of the modules.

### Identification of Sequence Variants

For genes of interest, we used the Sanger Mouse Genomes Project SNP Query tool^1^ to identify sequence variants (SNPs, Indels, and structural variants) between the C56BL/6J and DBA/2J mouse strains, the parental strains of the BXD panel. Variant consequences are predicted using the Ensembl Variant Effect Predictor.

### Statistics

Statistics were completed in R and figures were generated using the R packages ggplot2 (cognitive performance, reserve/resilience plots), corrplot (correlation matrices), and qtl2 (QTL plots), or Microsoft Excel (module enrichment plots). Significance thresholds were set to alpha = 0.05 and adjusted for multiple corrections as specified.

## Results

### Working Memory Is Heritable, Polygenetic, and Regulated by Cellular Metabolism Transcriptomic Pathways

**FIGURE 2:**
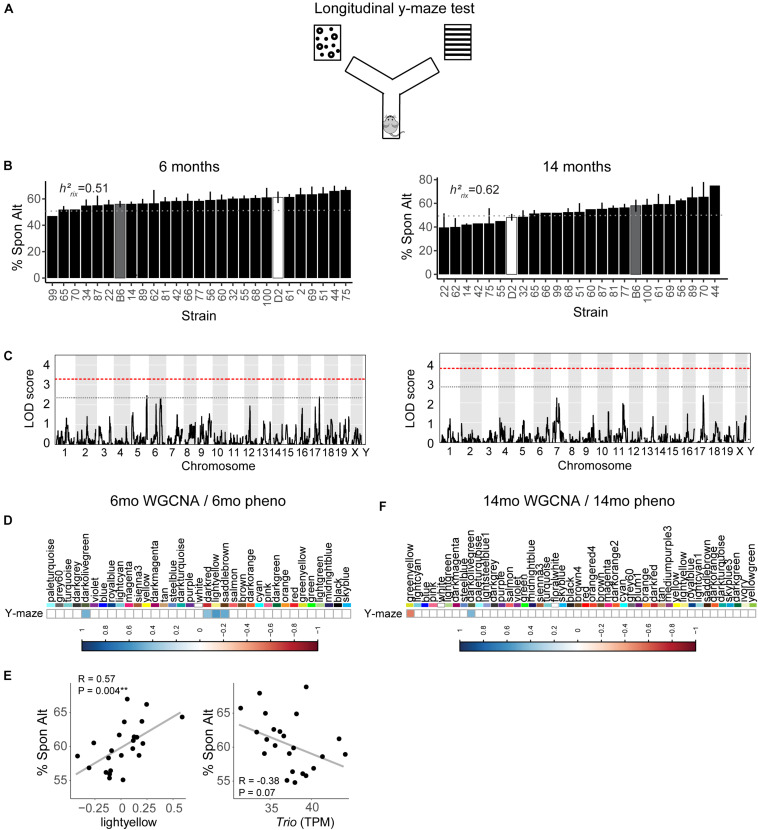
Variation in working memory is heritable in early adulthood (6 months) and middle age (14 months) but is not significantly controlled by a single genetic locus. **(A)** We used the y-maze as a test of working memory in B6-BXD mice at both 6 months and 14 months. In this version of the y-maze test, we used visual cues placed outside of the maze and allowed the mouse to freely explore each arm. Entering each of the three arms in succession was considered a successful spontaneous alternation (“Spon Alt”). **(B)** Performance on the y-maze test is heritable (*h*^2^_*RIx̄*_ = 0.51 at 6 months and 0.62 at 14 months). **(C)** Quantitative trait locus mapping indicated that no single genetic locus contributed significantly to performance on the y-maze at either age, suggesting that working memory is a polygenetic trait. **(D)** The relationship between each WGCNA module eigengene expression and cognitive function at 6 months was assessed using Pearson correlations. Significant correlations are represented by blue (positive correlation) or red (negative correlation) shading, with color intensity corresponding with correlation strength. Four WGCNA modules were associated with y-maze at 6 months: darkolivegreen, darkred, lightyellow, and saddlebrown. **(E)** Expression of the lightyellow module was significantly positively associated with working memory at 6 months (*R* = 0.57, *p* = 0.004); however, expression of the hub gene of this module *Trio*, was not significantly associated with working memory (*R* = −0.38, *p* = 0.07). **(F)** Two modules at 14 months were significantly correlated to performance on y-maze at 14 months: greenyellow and darkolivegreen. *QTL significance thresholds: red line, alpha = 0.05; black line, alpha = 0.33.*

We assessed hippocampus-dependent working memory by measuring spontaneous alternations in y-maze at both 6 months and 14 months of age (Figure 2A). A majority of strains had a mean performance above chance (i.e., 50% spontaneous alternations) at 6 months, demonstrating that these mice were generally able to perform this task at baseline. We also performed one-sample *t*-tests within each strain (with *n* > 2) to identify which strains performed statistically significantly above chance. Several strains did not perform significantly above chance levels (CI = 99%; see Supplementary Table S2 for a summary of these one-sample *t*-tests), though this is likely due to reduced power given the relatively low number of biological replicates per strain required for such a study using a genetic reference panel. By 14 months, two of the 25 strains tested (B6-BXD62 and B6-BXD14) had a mean (±standard error) performance below chance, indicating vulnerability to cognitive impairment by middle age, and all but three strains (B6-BXD56, B6-BXD77, B6-BXD81; see Supplementary Table S3 for a summary of these *t*-tests) were performing statistically equal to chance by one-sample *t*-tests. We then calculated heritability, *h*^2^_*RIx̄*_, to determine the proportion of trait variation that is genetically controlled. Heritability of working memory improved with age (*h*^2^_*RIx̄*_ = 0.51 at 6 months vs. 0.62 at 14 months – or 51% and 62% of the variance at 6 and 14 months, respectively, may be attributed to genetic factors) (Figure 2B and Table 1). Such high heritability of working memory implies genetic control; to identify potential genetic drivers of these traits, we performed QTL mapping. QTL mapping revealed no single locus controlling a significant proportion of the variance on performance on y-maze at either 6 months or 14 months of age (Figure 2C), or combined with age as a covariate (data not shown). We then employed RNA sequencing to identify mechanisms underlying the variation in working memory.

We performed weighted gene co-expression network analysis (WGCNA) from 6 to 14 months RNA-seq data to identify clusters of genes (i.e., modules) that have highly correlated expression across our population. We calculated Pearson’s correlations for expression of each module (i.e., expression of the module eigengene) with cognitive function at concurrent time points (i.e., 6 months RNA expression to 6 months phenotypes and 14 months RNA expression to 14 months phenotypes) to determine which modules were most likely underlying working memory. To characterize each module, we identified the hub gene most highly connected within the module, and performed gene ontology (GO) enrichment to describe biological pathways and molecular functions associated with each module. Four WGCNA modules were significantly positively associated with strain differences in working memory performance at 6 months (Figure 2D). These modules were associated with functions encompassing cellular metabolism (lightyellow; *R* = 0.62, nominal *p*-value = 0.004; 23 significant GO terms by FDR < 0.05), RNA and protein localization (saddlebrown; *R* = 0.56, *p*-value = 0.02; 9 significant GO terms by FDR < 0.05), DNA stability (darkred; *R* = 0.47, *p* = 0.049; 36 significant GO terms by FDR < 0.05), and receptor recycling (darkolivegreen, *p*-value = 0.02; 9 significant GO terms by FDR < 0.05) (see Table 2 for hub genes and top GO significant terms by enrichment ratio for these modules). The most strongly correlated module, lightyellow, was regulated by its hub gene, *Trio*, a guanine nucleotide exchange factor. *Trio* is important in neuronal development and synapse function and has been previously associated with cognitive ability: mutations in *TRIO* lead to intellectual disability in humans (Ba et al., 2016; Pengelly et al., 2016), and hippocampal and cortical knockout of *Trio* leads to impaired learning in memory in mice (Zong et al., 2015). There is one missense variant in *Trio* in the DBA/2J genome compared to the C57BL/6J genome. This variant (Chr 15:27752684, a/c) is within the coding region of *Trio* and results in a change from a valine to glycine and has a SIFT (Sorting Intolerant From Tolerant) score of 0, indicating a deleterious effect on protein expression. This indicates that there is likely differential function of *Trio* across our B6-BXD cohort that may disrupt the larger lightyellow network, associated cellular metabolism pathways, and ultimately working memory ability (Figure 2E).

**TABLE 2:** Top GO terms and hub genes for 6 months gene modules significantly associated with 6 months cognitive function.

WGCNA Module	Associated phenotype	Top GO term (top enrichment score with FDR < 0.05)	Hub gene
darkolivegreen	Y-maze	positive regulation of receptor recycling	*Krt2*
darkred	Y-maze	DNA topoisomerase type I activity	*Cfap20*
lightyellow	Y-maze	heterocyclic compound binding	*Trio*
saddlebrown	Y-maze	establishment of protein localization to Golgi	*Smim10l2a*
black	CFA	regulation of cell differentiation	*Qars*
lightgreen	CFA	positive regulation of protein targeting to mitochondrion	*Mccc1*
paleturquoise	CFM	ubiquitin-ubiquitin ligase activity	*Herc3*
red	CFM	cellular response to stress	*Szt2*

At 14 months, working memory was correlated with the two modules, only one of which (greenyellow) was significantly enriched for GO terms (*R* = −0.48, *p*-value = 0.008; 31 significant GO terms by FDR < 0.05; Figure 2F and Table 3). This module was enriched for genes associated with DNA binding and metabolism pathways, and is regulated by its hub gene, *Ptpn6*–a protein tyrosine phosphatase. *Ptpn6* has not previously been associated with learning and memory, though protein stability and specifically protein phosphatases are important for learning, memory, and synaptic functions (Graff et al., 2010). These data suggest that disrupted nucleic acid metabolism may be associated with poorer cognitive function in aging.

**TABLE 3:** Top GO terms and hub genes for 14 months gene modules significantly associated with 14 months cognitive function.

WGCNA Module	Associated phenotype	Top GO term (top enrichment score with *p* < 0.05)	Hub gene
darkolivegreen	Y-maze	None	*Usp2*
greenyellow	Y-maze	DNA recombination	*Ptpn6*
plum1	CFA	retrograde vesicle-mediated transport, Golgi to ER	*Fam160b2*
tan	CFA	regulation of transcription, DNA-templated	*Nkrf*

### Contextual Fear Acquisition and Memory Are Not Significantly Regulated by Specific Genomic Loci

**FIGURE 3:**
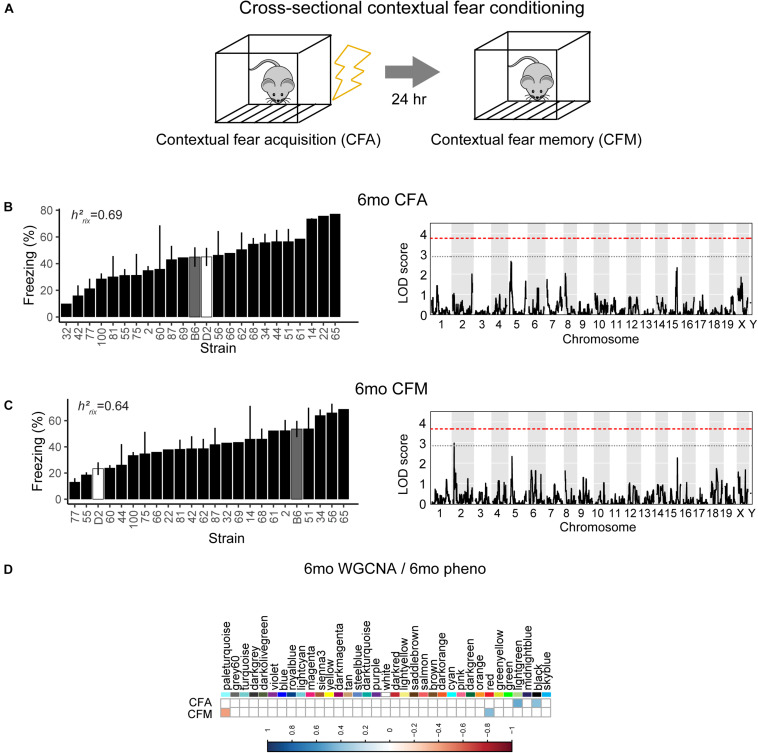
Variation in short- and long-term memory is heritable and not controlled by a single genetic mechanism at 6 months of age. **(A)** B6-BXD mice underwent contextual fear conditioning to assess short- and long-term memory. Mice received four mild footshocks and were tested for contextual memory by measuring freezing 24 h later. **(B)** Contextual fear acquisition, or freezing during the postshock 4 (PS4) interval, was heritable (*h*^2^_*RIx̄*_ = 0.69) at 6 months. QTL mapping revealed that no locus was significantly associated with performance on contextual fear acquisition. **(C)** Contextual fear memory performance is also highly heritable (*h*^2^_*RIx̄*_ = 0.64); however, QTL mapping failed to identify significant loci controlling contextual fear memory. **(D)** Four WGCNA modules were associated with contextual fear conditioning traits at 6 months: CFA was significantly associated with the lightgreen and black modules’ expression. CFM was significantly associated with the paleturquoise and red modules’ expression. *QTL significance thresholds: red line, alpha* = *0.05; black line, alpha* = *0.33.*

To assess hippocampus-dependent acquisition of contextual fear memory in young adulthood, animals underwent contextual fear conditioning (Figure 3A). Acquisition of contextual fear conditioning (CFA) was highly heritable, as measured by comparing within strain versus across strain variability using percent freezing during the interval following the fourth shock (postshock 4 interval; *h*^2^_*RIx̄*_ = 0.69 at 6 months) (Figure 3B). The high degree of heritability indicates strong genetic control, therefore we performed QTL mapping for CFA (using mean freezing in the final postshock interval) and did not identify any locus significantly associated with this trait. Similarly, although contextual fear memory was also highly heritable (CFM; *h*^2^_*RIx̄*_ = 0.64 at 6 months), we did not identify any loci significantly associated with contextual fear memory, suggesting polygenetic control of long-term contextual fear memory at 6 months (Figure 3C).

### Contextual Fear Acquisition and Memory in Young Adulthood (6 Months) Are Associated With Gene Networks Involved in Cell Metabolism and Gene Transcription

Here, we turned to WGCNA to identify gene co-expression networks underlying performance on CFA and CFM (Figure 3D). CFA at 6 months was significantly associated with two WGCNA modules (Figure 3D). The module with the strongest positive association with CFA, lightgreen (*R* = 0.34, *p*-value = 0.01), was significantly enriched for 39 GO terms encompassing cellular metabolism and gene transcription (FDR < 0.05). Similarly, the red module was significantly positively associated with CFM (*R* = 0.43, *p*-value = 0.04) and was also significantly enriched for 50 GO terms encompassing gene transcription and protein synthesis pathways (FDR < 0.05; See Table 2 for top GO terms and hub genes for these modules). These data highlight the requirement of synapse remodeling and protein expression changes in learning and memory consolidation in a task such as fear conditioning (Alberini and Kandel, 2015).

### Wide Variation in Cognitive Performance of 14 Months (Middle-Aged) Mice Is Regulated by Networks Involved in Gene Transcription

**FIGURE 4:**
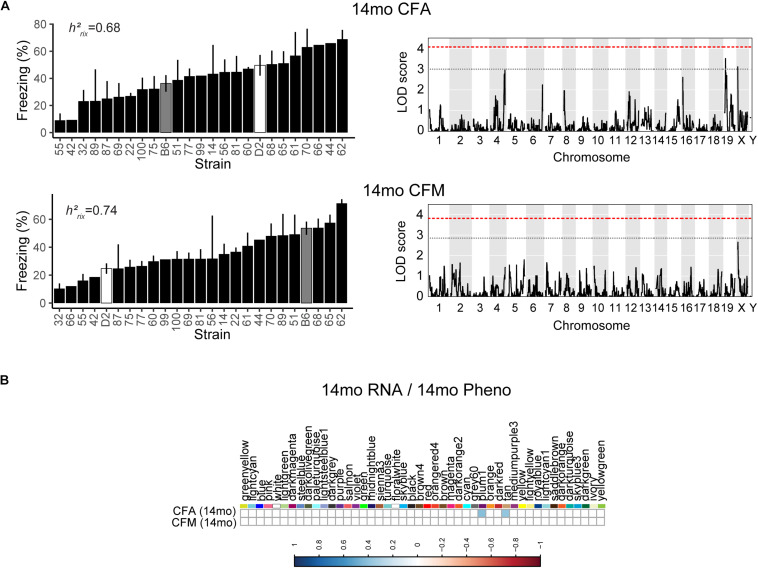
Contextual fear conditioning performance in middle age is also highly heritable and polygenetic, and is associated with neuronal function. **(A)** Contextual fear acquisition and contextual fear memory were both highly heritable at 14 months (CFA: *h*^2^_*RIx̄*_ = 0.68, CFM: *h*^2^_*RIx̄*_ = 0.74), though we did not identify any significant locus associated with CFA or CFM in middle age. **(B)** We identified two WGCNA modules that were associated with CFA, but not CFM, at 14 months.

We then looked at performance on contextual fear conditioning in middle age (14 months). Although performance on both CFA and CFM was highly heritable at 14 months (CFA: *h*^2^_*RIx̄*_ = 0.68 at 14 months; CFM: *h*^2^_*RIx̄*_ = 0.74 at 14 months; Table 3), QTL mapping revealed no genome-wide loci associated with either CFA or CFM at 14 months (Figure 4A), again hinting at the highly polygenetic nature of these traits. To interrogate the molecular underpinnings of phenotypic variation of contextual fear conditioning in middle age, we again turned to WGCNA. Here, two modules were significantly positively correlated with CFA (tan: *R* = 0.41, *p*-value = 0.049, 37 significant GO terms by FDR < 0.05; plum1: *R* = 0.40, *p*-value = 0.03, 1 significant GO term by FDR < 0.05), and no modules correlated with CFM in middle age (Figure 4B). Similar to modules underlying CFA and CFM at 6 months, networks important in gene transcription and biosynthesis (tan module), and protein transport (plum1 module) were important in regulating CFA at 14 months (see Table 4 for top GO terms and hub genes for these modules).

**TABLE 4:** Top GO terms and hub genes for 6 months gene modules significantly associated with later (14 months) cognitive function and/or decline.

6 months WGCNA Module	Associated phenotype	Top GO term (top enrichment score with *p* < 0.05)	Hub gene
blue	14 months CFA	receptor localization to synapse	*Glg1*
darkgreen	14 months CFA	central nervous system myelination	*Cnp*
darkgray	CFA decline	n/a	*Klhl23*
darkmagenta	14 months CFM, CFM decline	binding	*Eda*
darkorange	14 months CFA, CFA decline	GPI-anchor transamidase activity	*Ngb*
darkturquoise	CFM decline, CFA decline	response to cold	*Cnrip1*
greenyellow	14 months CFA	regulation of transcription involved in meiotic cell cycle	*Kat2b*
orange	CFM decline	U1 snRNP binding	*Otub2*
pink	14 months CFA	structural constituent of ribosome	*Eml5*
tan	14 months CFA, CFM decline, CFA decline	cellular localization	*Nkrf*
turquoise	14 months CFA, CFA decline	syntaxin binding	*Mdfic*
white	CFA decline	protein binding	*Gm22291*
yellow	14 months CFA	rRNA processing	*Gria1*

### Age-Related Cognitive Decline Is Polygenetic and Predicted by 6 Months Neuronal Gene Networks

One strength of our B6-BXD model of cognitive aging is that each strain has a stable, reproducible genome and as such may be resampled to assess individual strain differences age-related cognitive decline. In general, in our B6-BXD population, cognitive performance declined with age. We found a significant main effect of age on y-maze by ANOVA (*F* = 18.54, *p* < 0.001), though there was no significant effect of age by ANOVA at the population level on contextual fear memory traits (CFA: *F* = 0.422, *p* = 0.52; CFM: *F* = 2.48, *p* = 0.11). We then calculated a “decline score” for each strain on each trait: this was done by subtracting strain average performance at baseline (6 months, adult) from performance at 14 months (middle-aged).

**FIGURE 5:**
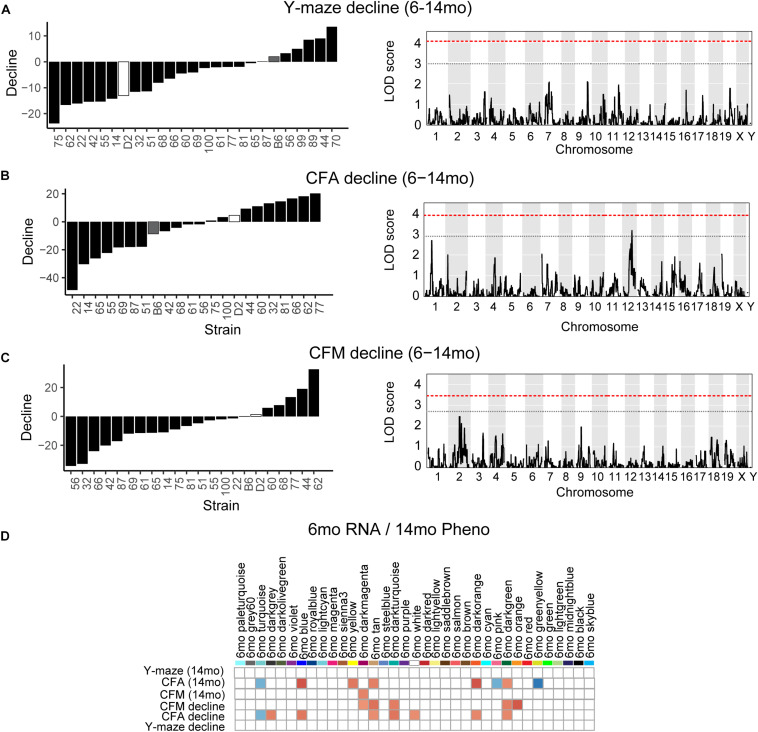
Cognitive decline is polygenetic and may be predicted by early life gene co-expression networks. **(A)** B6-BXD strains generally declined on working memory between 6 and 14 months of age, though there was wide variation in degree of change. QTL mapping revealed no significant locus regulating this decline. **(B,C)** B6-BXD strains showed a wide range in change in performance on contextual fear conditioning between 6 and 14 months. Again, no single locus was associated with decline on either contextual fear acquisition or memory. **(D)** We identified several WGCNA modules whose expression at 6 months were significantly associated with later performance and/or decline on contextual fear acquisition and memory.

The majority of strains (17/24) showed poorer working memory at 14 months compared to 6 months (Figure 5A). However, only about half of the strains exhibited declined on CFA and memory, 12/22 and 14/22, respectively with age, and some performed better at mid-life (Figures 5B,C). These data indicate strong individual differences in cognitive decline that may be genetically controlled and resolved through genetic mapping. We thus performed QTL mapping on cognitive decline scores generated for working memory, CFA and CFM, and did not identify any loci significantly associated with cognitive decline on any of the traits measured. These data suggest that, like in the human population, complex polygenetic interactions determine the rate of cognitive decline in our mice, where no single locus had a sufficiently large effect size for us to detect given our sample size (Figures 5A–C). This finding is another indication of the translational relevance of our model; an additional advantage this model has over human populations is that we were then able to turn to longitudinal hippocampal brain transcriptomic co-expression networks to interrogate the molecular networks underlying decline. We observed several strong associations between gene expression in earlier adulthood (6 months) and later performance and decline on contextual fear conditioning (14 months), with 13 modules’ expression at 6 months significantly correlated with performance and/or decline on one or more of the measured cognitive domains (Figure 5D). These modules were enriched largely for neuronal pathways and gene transcription. In particular, myelination and synaptic function at 6 months were strongly associated with later cognitive function and decline (see Table 4 for top GO terms and hub genes for each module). This suggests that maintenance of cognitive function through middle age may be particularly regulated by both neuronal function and gene transcription/protein stability. More broadly, the strong relationships between hippocampal gene expression in earlier adulthood and mid-life cognitive performance suggests that age-related cognitive decline is sensitive to early life molecular processes, and that interventions to prevent age-related cognitive decline should target these early perturbations in relevant gene networks.

### Characterization of Cognitive Reserve and Resilience in the B6-BXDs

**FIGURE 6:**
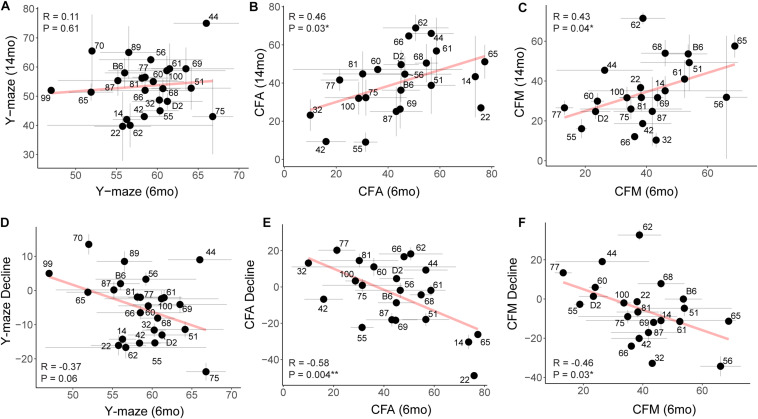
Early performance on contextual fear memory, but not y-maze, predicts later performance. **(A)** Pearson’s correlation indicates no significant relationship between early (6 months) and later (14 months) performance on the y-maze test (*R* = 0.11, *p* = 0.61). Gray dashed lines in the y-maze plot indicate 50% spontaneous alternations, or chance performance. Performance below 50% indicates cognitive impairment on this test. Error bars represent standard error. **(B,C)** Pearson’s correlation indicates that early (6 months) performance on contextual fear conditioning predicts later (14 months) performance by both CFA and CFM. Better performance at 6 months predicts better relative performance at 14 months (*R* = 0.46, *p* = 0.03 for CFA; *R* = 0.43, *p* = 0.04 for CFM). **(D–F)** Pearson’s correlations indicate that higher performance in early adulthood (6 months) is associated with greater decline by midlife (14 months), particularly in short- and long-term memory (*R* = −0.37, *p* = 0.06 for y-maze; *R* = −0.58, *p* = 0.004 for CFA; *R* = −0.46, *p* = 0.03 for CFM). ^∗^*p* < 0.05; ^∗∗^*p* < 0.01.

To assess whether higher baseline cognitive function results in better cognitive function in aging (i.e., early cognitive reserve conferring protection against later decline) (Cook and Fletcher, 2015; Lesuis et al., 2018; Bettcher et al., 2019; Walhovd et al., 2019; Zahodne et al., 2019), we compared baseline cognitive function at 6 months to later cognitive performance at 14 months. To do this, we plotted later (14 months) performance as a function of earlier (6 months) performance and calculated the Pearson’s R to assess whether there was a correlation between early life and midlife cognitive function. We saw no association between early y-maze performance and later y-maze performance (Figure 6A; *R* = 0.11, *p* = 0.61), suggesting that better performance at young ages on this task does not confer protection against decline in working memory. However, better performance on contextual fear CFA and CFM in adulthood predicted superior memory performance on CFM in middle-aged mice (Figures 6B,C; CFA: *R* = 0.46, *p* = 0.03; CFM: *R* = 0.43, *p* = 0.04). These data suggest short-term and long-term memory performance later in life is protected to some degree by either having greater cognitive reserve evident in early adulthood or cognitive resilience protecting against decline in midlife. To further clarify this, we next asked whether higher baseline (6 months) function protected against cognitive *decline.* In fact, higher performance at 6 months generally resulted in greater decline by 14 months (Figures 6D–F), which may simply reflect the mathematically greater potential for decline in baseline high-performers, or it may reflect the ability of greater cognitive reserve to protect against cognitive *impairment* even when an individual experiences cognitive decline from their own baseline.

#### Working Definition of Cognitive Reserve and Resilience

**FIGURE 7:**
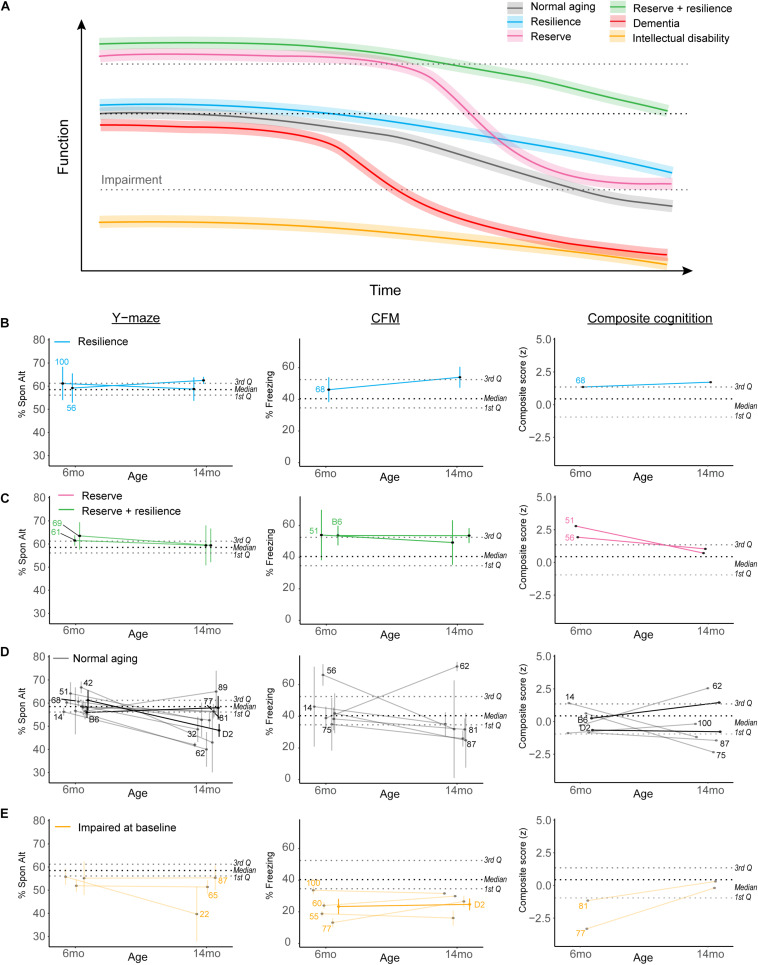
Complex cognitive trajectories in aging may be modeled in the B6-BXD mice. **(A)** Visualization of idealized data exemplifying cognitive functional decline in normal aging (gradual decline after midlife; black line), dementia (rapid decline to impairment in midlife; red line), cognitive reserve (higher baseline; pink line), cognitive resilience (slower decline; blue line), or both cognitive reserve and resilience (green line). Individuals with intellectual disability or impaired performance at baseline (orange line) are typically excluded from human studies in cognitive aging. **(B,C)** We identified strains displaying suggestive reserve and/or resilience on individual cognitive measures; these strains remain only suggestively in their categories because, though strain averages fall within the limits of our definitions, within-strain variability exceeded our margins of error in every case. For individual traits, B6-BXD100 and B6-BXD56 were suggestive resilient strains, and B6-BXD69 and B6-BXD61 were suggestive reserve + resilient strains in y-maze. B6-BXD68 was a suggestive resilient strain, and B6-BXD51 and B6 homozygotes were suggestive reserve + resilient strains in CFM. Right panels: We also calculated a composite score for cognitive performance across three traits (y-maze, CFA, and CFM) by summing the z-score for each trait within each strain. B6-BXD68 was a suggestive resilient strain, and B6-BXD51 and B6-BXD56 were suggestive reserve strains. **(D)** Strains that had baseline (6 months) performance below the 3rd Quartile (i.e., no cognitive reserve), midlife (14 months) performance below the median (i.e., insufficient cognitive reserve), or a greater than average rate of decline (i.e., no cognitive resilience) were considered “normal agers”. For **(B–E)**, color scheme is as in **(A)**. Black dashed line is median, gray dashed lines are 1st and 3rd quartile. For ease of visualization, each category of aging trajectory was visualized separately. Strains with *n* < 2 at either time point were excluded from categorization and visualization. **(E)** Strains with baseline performance below the 1st quartile were considered impaired.

Because our population-level data hinted at a role for cognitive reserve or resilience protecting against midlife cognitive decline, we sought to objectively operationally define cognitive reserve and resilience using our B6-BXD population and identify individual strains which may represent these cognitive aging strategies. In Figure 7A, we demonstrate hypothetical cognitive trajectories for normal aging (black line), dementia (red), and cognitive aging in populations with cognitive reserve (pink) and/or resilience (blue, green). In these latter cases, cognitive decline is buffered by cognitive reserve and slowed by cognitive resilience. We expected that a small number of B6-BXD strains might exemplify either cognitive reserve or resilience by middle-age given the variation in cognitive decline we observed, so we first objectively defined reserve and resilience, preregistered these definitions, and then tested whether any strains in our population met these criteria. This definition and identification of potential “strains of interest” will be particularly useful for future studies – both using the BXD panel to more deeply characterize resilience and reserve within these strains, and also to inform human studies toward a more mechanistic definition of “reserve” and “resilience” based in the biological processes underlying these characteristics.

Cognitive reserve is commonly defined as higher cognitive function at baseline (Montine et al., 2019; Figure 7A, pink and green lines), which allows for more cognitive flexibility and buffering against cognitive decline. For our definition, we considered strains in the top quartile to display cognitive reserve. Higher cognitive function at baseline allows for “reserve capacity” to buffer against any cognitive decline, but does not necessarily stem cognitive decline. However, because cognitive reserve necessarily should protect against later cognitive *impairment* (regardless of decline from baseline), and because our terminal time point (14 months) is in middle age for mice, we also required strains with cognitive reserve to still function above the median population performance at 14 months. Ideally, cognitive reserve and resilience would manifest globally, or protect against impairment across cognitive domains (i.e., working, short-term, and long-term memory in our dataset), so in addition to individual cognitive traits, we also calculated a composite score for cognitive function by calculating z-scores for each strain for each cognitive trait and timepoint, and summing these scores to achieve a composite score.

Resiliency to cognitive decline is commonly defined as a relatively slow rate of decline over time (e.g., the annual rate of change in humans; Figure 7A green and blue lines) (Montine et al., 2019). Though performance across the spectrum (from “good” performers to “poor” performers at baseline) may be stable across time and thus display a type of resilience to decline, we required that strains that displayed “true” resilience to be relatively good performers at baseline (i.e., performing above the median as indicated by the black dashed line in Figures 7B–E) and to decline more slowly than average. Initially, we defined slow decline as “no significant difference from 6 to 14 months performance.” However, this criterion proved too permissive, as few individual strains exhibited significant decline after adjusting for multiple comparisons (see previous sections). Our final definition of cognitive resilience required strains start above median population performance (indicated in Figures 7B–E as the middle black dashed lines) and have an “annual rate of decline” (or decline slope) slower than average.

Finally, in identifying strains that met these definitions, we required that strain average ± strain standard error fit within these criteria. Ultimately, these strains fell along a continuum of cognitive performance and decline, and due to the within-strain variance, no single strain exemplified true reserve or resilience based on our working definition. We identified strains that may potentially fit these definitions given more thorough characterization and a higher sample size; these “suggestive” strains may be promising strains to investigate with a higher sample size to identify molecular signatures of reserve and/or resilience and are delineated in Figures 7B–E. For ease of visualization, we have plotted resilience, reserve and reserve/resilience, normal aging, and impaired strains in separate plots. Strains with an *n* < 2 at any given timepoint were excluded from these visualizations, as we were unable to assess within-strain variance. Strains meeting suggestive criteria for reserve and resilience included: for resilience, B6-BXD100 and B6-BXD56 for working memory and B6-BXD68 for long-term memory and in our composite cognitive score (Figure 7B, blue lines); for reserve, B6-BXD51 and B6-BXD56 with composite cognitive score (Figure 7C, pink lines); for reserve + resilience, B6-BXD61 and B6-BXD69 for working memory, and B6 and B6-BXD51 in contextual fear memory (Figure 7C, green lines). Most strains fell within “normal aging” parameters (that is, starting within the middle quartile ranges at baseline and/or not meeting our reserve/resilience criteria). In Figure 7E, we identified several strains (orange lines) whose baseline performance fell below the first quartile (lower gray dashed lines in Figures 7B–E), suggesting baseline impairment.

Further characterization of these strains to specifically test for and more deeply characterize cognitive reserve and resilience will be necessary, including aging mice much longer (e.g., to 22 months or older), though we demonstrate here that the B6-BXD population is a powerful tool to begin understanding the nature of reserve and resilience and to identify the molecular networks underlying these traits.

## Discussion

Genetics of cognition, cognitive decline, and cognitive reserve are highly complex and difficult to study in humans. However, as we make strides in improving lifespan, increasing cognitive longevity should become a priority in order to maximize quality of life in old age. Understanding the molecular mediators of baseline cognitive function, cognitive reserve, resiliency and susceptibility with regards to age-related cognitive decline and identification of novel pharmacological targets/pathways regulating cognitive health may allow cognitive health span to catch up to lifespan improvements afforded by modern medicine. To achieve these goals, we first need to identify models that can best address these questions. Here, we have utilized a novel model of age-related cognitive decline to extract genetic mediators of normal cognitive function and age-related decline. Because our B6-BXD population is a recombinant inbred backcross rather than a homozygous BXD population, the result is enrichment for identifying B6 effects on phenotypes by genetic mapping, and a loss of detecting recessive D2 effects. However, given the great interest in identifying genetic factors harbored by the B6 strain that confer documented resilience against cognitive impairment compared to D2 (Neuner et al., 2019), we hypothesized we would identify genetic resilience mechanisms within our population.

### We Observed Heritable Variation in Cognitive Tasks and Age-Related Cognitive Decline

The heritability estimates (*h*^2^_*RIx̄*_) of working, short- and long-term memory ranged from 0.51 to 0.74, indicating that these traits are strongly genetically controlled. However, QTL mapping identified no single significant peak associated with any trait, meaning that no individual genetic locus accounted for a substantial proportion of the variance on these traits as could be detected by our sample size. These data indicate that cognitive function is polygenetic and controlled by many variants with small effect sizes, as we did not identify additional loci associated with later cognitive function or cognitive decline. This is not surprising, given recent GWAS in humans have found hundreds of SNPs associated with cognitive function and other highly complex traits (Davies et al., 2018). Given the importance of understanding the molecular contributors to cognitive function and reserve against decline with age, though, we sought to develop alternative approaches to identifying genes and pathways that mediate cognitive function and promoting cognitive reserve and resilience.

### Prioritization of Molecular/Genetic Candidates of Cognitive Function and Decline in Adulthood

To complement our QTL mapping and to identify gene networks underlying complex cognitive traits, we conducted molecular experiments to identify target genes associated with cognition. We performed RNA sequencing followed by weighted gene co-expression analysis (WGCNA), and measured the association of WGCNA modules and individual genes with level of cognitive performance and decline. We identified pathways and highly interconnected “hub” genes from our WGCNA modules, which are functionally important within gene networks and may represent candidate genes and mechanisms underlying “normal” cognitive aging. Given that cognitive function and decline are incredibly complex and are affected by a wide range of factors, we hypothesized that associated gene networks would be more biologically relevant than single candidate genes, and provide more insight to the mechanisms underlying cognitive function and decline. These gene networks, in turn, may be manipulated therapeutically by targeting their hub genes with the goal of enhancing cognitive function in aging. Perhaps unsurprisingly, the modules that were most significantly associated with cognitive function in adulthood were enriched for GO terms associated with cellular metabolism and transcription/translation, highlighting the importance of physical remodeling of synapses in learning and memory through regulation of gene transcription and protein expression/localization (Alberini and Kandel, 2015). Notably, evidence from postmortem human brain studies suggest that synapse or dendritic spine remodeling is a primary neurobiological mechanism of cognitive resilience to aging and Alzheimer’s disease pathology (Boros et al., 2017, 2019).

As with baseline cognitive function, we also identified gene networks that primarily included neuronal, transcription/translation, and cellular metabolism functions as underlying cognitive decline. This was in contrast to our previous analyses of our genetically diverse population of mice with familial Alzheimer’s disease mutations (AD-BXDs; Neuner et al., 2019), where we observed largely neuroinflammatory pathways as underlying AD-related cognitive decline. Our AD-BXD and B6-BXD populations did have pathways enriched in neuronal function in common. These findings suggest that an individual’s risk for disease-related cognitive decline versus “normal” aging mechanisms may have some common elements (e.g., neuronal function); though we also observe an interesting divergence in pathways, where disease may be regulated by neuroinflammatory processes, and normal aging may be regulated by cell metabolism and maintenance of proper gene expression and nucleic acid stability.

Intriguingly, we identified the strongest and most numerous gene co-expression network-trait associations between 6 months gene expression and later cognitive function and decline. This indicates that variation in gene expression in early adulthood may likely determine cognitive decline, rather than later gene expression perturbations being most significant to underlying in cognitive decline. In addition to implying that interventions must happen early in order to curb age-related cognitive decline, this also highlights the value in collecting behavioral data and brain molecular information at early time points, a process which is impossible in human studies. Thus, to understand the molecular mechanisms of cognitive aging, we need to focus on animal models such as our B6-BXD population where we are able to sample timepoints across the lifespan.

### Identifying Strains Characterized by Cognitive Reserve and Resilience in the B6-BXD Population

Finally, we assessed whether we observe cognitive reserve and resilience in our population of mice. In human literature, cognitive reserve and resilience are inconsistently defined, which has contributed to a general lack of focus in understanding of the mechanisms underlying these processes. For example, many studies use “years of education”–or related measures such as being multilingual or having a cognitively engaging occupation–as a proxy for cognitive reserve. Defining cognitive reserve in this way is problematic for multiple reasons: namely, though the two are often correlated, we do not believe that socioeconomic opportunity is intrinsically required for cognitive reserve. Additionally, to study cognitive reserve in animals–and the genetic basis thereof–we also cannot rely on external factors such as education that are inapplicable to animals. Cognitive reserve is likely plastic and may be enhanced by environmental enrichment in both humans and animals – an additional goal of our laboratory will be to evaluate individual differences in how environmental enrichment may enhance cognitive reserve.

Cognitive resilience has also been inconsistently defined in the literature and often implies resilience to disease-related processes, such as atrophy or neurodegenerative disease pathologies. We sought to define cognitive resilience behaviorally, and in the future will extend this definition to identify anatomical, cellular, molecular signatures of cognitive resilience to maximize translatability to human studies (Bettcher et al., 2019). Namely, we required that cognitive resilience was characterized by slow (or non-existent) cognitive decline over time. Our late-life timepoint for measuring cognitive function was 14 months, which approximates middle age. Although 14 months is not considered “aged” for these strains of mice, this does raise an important point in the context of translatability of these measures: in human studies, participants are typically enrolled in mid-life when some degree of cognitive decline may have already occurred, even if “control” participants are still performing cognitive tasks well. A strength of our mouse model is the ability to sample both cognitive and molecular data at early time points to relate early changes with mid- and late-life cognitive function. Our observation that gene perturbations in early adulthood (i.e., 6 months) may be more important in regulating cognitive decline than later transcriptomic changes indicates that human studies may be starting too late to fully characterize reserve and resilience trajectories and mechanisms. On the other hand, our study likely ended too early (middle age) and thus we were unable to observe robust effects of cognitive resilience. It is likely that to truly identify cognitive resilience to age-related decline, we will need to observe cognitive function through late life, or to at least a 22–24 months timepoint.

Ultimately, we established a stringent set of criteria to operationally define cognitive reserve and resilience in the B6-BXD population that may be extended to other animal models as well as human studies. First, we required that animals with cognitive reserve have baseline functioning in the upper quartile. We expected that cognitive reserve would vary based on cognitive domain – that is, strains could display cognitive reserve as measured by one or a subset of tasks. We also expect that cognitive reserve functions to protect against cognitive impairment with age, so we also required that strains would still be performing at or above median performance by 14 months.

We expected the effects of cognitive resilience to be global and exhibit protection against decline across cognitive domains. In this case, we required strains with cognitive resilience to start at or above the median population performance. We also required that there be no significant decline in cognitive function between 6 and 14 months. To fit within these criteria, strains would have to perform on average, ±standard error, within their category. In our population, we did not observe sufficient evidence to identify true exemplars of cognitive reserve or resilience, though we were able to identify multiple strains that *may* represent either cognitive reserve, resilience or both. Thus, with our criteria, we will need additional biological replicates per strain, and likely additional strains, to fully capture cognitive reserve and resilience. This highlights the main advantage of our mouse model of normal aging: because each strain has a replicable genome, we are able to add biological replicates to more deeply characterize any strain or trait of interest. In the future, we will expand our studies to include more strains, more animals per strain, and extended timepoints in order to capture the a more precise picture of within-strain performance and a full range of cognitive performance and decline. This will allow us to identify molecular signatures of cognitive reserve and resilience in our B6-BXD population, and – most importantly – assess the translational potential of these findings to human studies. By establishing objective definitions of cognitive reserve and resilience, and by identifying mouse models of these traits, we will be able to inform human studies of candidate molecular mechanisms for successful cognitive aging that may, in turn, be used to develop therapeutics to prevent age-related cognitive impairment.

## Conclusion

Harnessing the underlying mechanisms of cognitive reserve and resilience will be a promising strategy to maintaining cognitive health until late life. Our understanding of the molecular underpinnings of reserve and resilience have been limited, but the development and usage of animal models of these processes, such as the B6-BXD recombinant inbred lines described herein, will provide an unprecedented opportunity to interrogate the early molecular mechanisms thereof and translate these findings to humans.

## Data Availability Statement

The raw RNA-seq data is also associated with earlier publications and, as such, has been previously uploaded to GEO (Accession Numbers GSE101144, GSE119215, and GSE119408).

## Ethics Statement

The animal study was reviewed and approved by the Institutional Animal Care and Use Committee (IACUC) at The University of Tennessee Health Science Center.

## Author Contributions

SN, KO’C, and CK conceived of and designed the experiments. SN conducted the behavioral experiments. AD, NH, SN, JGZ, VP, LD, TH, JH, KO’C, and CK conceived of and designed subsequent analyses, and assisted in data analysis and interpretation of results. AD, NH, SN, and JGZ performed the data analyses. AD and CK wrote the manuscript. All authors read and reviewed the final manuscript.

## Conflict of Interest

The authors declare that the research was conducted in the absence of any commercial or financial relationships that could be construed as a potential conflict of interest.

## Footnotes

1 https://www.sanger.ac.uk/sanger/Mouse_SnpViewer/rel-1505
