# Supplementary material for: Identifying Mechanisms of Normal Cognitive Aging Using a Novel Mouse Genetic Reference Panel
Source: Front Cell Dev Biol. 2020 Sep 11;8:562662. doi: 10.3389/fcell.2020.562662 (PMC7517308; doi:10.3389/fcell.2020.562662)
Supplement: Supplementary file 1 [file Table_1.PDF]

Supplementary table 1. Summary of animal numbers by strain represented in each behavioral assay and timepoint.

**Strain**	**Y-maze (6mo)**	**Y-maze (14mo)**	**CFC (6mo)**	**CFC (14mo)**
B6	12	4	6	9
B6xD2	13	14	11	12
B6xBXD2	7	0	5	0
B6xBXD14	4	2	2	2
B6xBXD22	4	3	1	2
B6xBXD32	4	3	1	3
B6xBXD34	10	0	11	0
B6xBXD42	5	1	4	1
B6xBXD44	2	1	2	1
B6xBXD51	7	4	3	4
B6xBXD55	6	1	4	2
B6xBXD56	5	2	3	2
B6xBXD60	4	1	2	3
B6xBXD61	6	5	1	5
B6xBXD62	3	3	2	3
B6xBXD65	9	8	1	8
B6xBXD66	2	1	1	1
B6xBXD68	19	10	9	10
B6xBXD69	4	5	1	4
B6xBXD70	1	2	0	3
B6xBXD75	9	5	3	6
B6xBXD77	12	10	2	10
B6xBXD81	8	5	3	5
B6xBXD87	6	3	3	3
B6xBXD89	2	2	0	2
B6xBXD99	1	1	0	1
B6xBXD100	6	4	2	4

Supplementary table 2. Summary of one-sample t-test results comparing y-maze performance at 6mo to chance performance (50% spontaneous alternations) in strains with n>2.

Strain	Average % Spon. Alt.	t-statistic	df	p-value
B6	56	2.47	11	0.015
B6xD2	61.23	2.59	12	0.012
B6xBXD2	63.43	2.72	6	0.017
B6xBXD14	56.25	3.67	3	0.017
B6xBXD22	55.75	1.66	3	0.098
B6xBXD32	60.25	6.87	3	0.003
B6xBXD34	54.9	1.05	9	0.16
B6xBXD42	58.4	3.16	4	0.017
B6xBXD51	64.14	2.92	6	0.013
B6xBXD55	60.33	4.57	5	0.003
B6xBXD56	59.2	1.45	4	0.11
B6xBXD60	59.5	2.50	3	0.044
B6xBXD61	61.5	5.15	5	0.002
B6xBXD62	56.67	0.66	2	0.290
B6xBXD65	51.89	0.70	8	0.253
B6xBXD68	60.68	4.99	18	4.72E-05
B6xBXD69	63.5	2.29	3	0.053
B6xBXD75	66.78	6.71	8	7.52E-05
B6xBXD77	58.5	5.56	11	8.57E-05
B6xBXD81	58.13	2.49	7	0.021
B6xBXD87	55.12	0.71	5	0.256
B6xBXD100	61.17	1.56	5	0.090

Supplementary table 3. Summary of one-sample t-test results comparing y-maze performance at 14mo to chance performance (50% spontaneous alternations) in strains with n>2.

**Strain**	**Average % Spon. Alt.**	**t-statistic**	**df**	**p-value**
B6	58.00	1.58	3	0.11
B6xD2	48.21	-0.64	13	0.73
B6xBXD22	39.67	-0.87	2	0.76
B6xBXD32	48.67	-0.24	2	0.59
B6xBXD51	52.75	0.37	3	0.37
B6xBXD61	59.40	1.09	4	0.17
B6xBXD62	40.00	-1.33	2	0.84
B6xBXD65	51.38	0.47	7	0.33
B6xBXD68	52.60	0.80	9	0.22
B6xBXD69	59.40	1.29	4	0.13
B6xBXD75	43.00	-0.54	4	0.69
B6xBXD77	56.50	2.10	9	0.03
B6xBXD81	56.20	3.05	4	0.02
B6xBXD87	55.33	1.02	2	0.21
B6xBXD100	58.75	1.73	3	0.09
